# Plasma Advanced Glycation End Products and Dicarbonyl Compounds Are Not Associated with Coronary Atherosclerosis in Athletes

**DOI:** 10.1249/MSS.0000000000003152

**Published:** 2023-04-04

**Authors:** KRISTIAN BERGE, VINCENT L. AENGEVAEREN, AREND MOSTERD, BIRGITTA K. VELTHUIS, MAGNUS N. LYNGBAKKEN, TORBJØRN OMLAND, CASPER G. SCHALKWIJK, THIJS M. H. EIJSVOGELS

**Affiliations:** 1 Department of Physiology, Radboud Institute for Health Sciences, Radboud University Medical Center, Nijmegen, THE NETHERLANDS; 2 Division of Medicine, Department of Cardiology, Akershus University Hospital, Lørenskog, NORWAY; 3 K.G. Jebsen Center for Cardiac Biomarkers, Institute of Clinical Medicine, University of Oslo, Oslo, NORWAY; 4 Department of Cardiology, Meander Medical Center, Amersfoort, THE NETHERLANDS; 5 Department of Radiology, University Medical Center Utrecht, THE NETHERLANDS; 6 Department of Internal Medicine, CARIM School for Cardiovascular Diseases, University Hospital Maastricht, Maastricht, THE NETHERLANDS

**Keywords:** CARDIOVASCULAR DISEASE, PLAQUE COMPOSITION, EXERCISE, ENDURANCE TRAINING, BIOMARKERS, CCTA

## Abstract

**Purpose:**

Coronary atherosclerosis is the leading cause of sudden death among athletes >35 yr old, but current cardiovascular risk prediction algorithms have not been validated for athletes. Advanced glycation end products (AGE) and dicarbonyl compounds have been associated with atherosclerosis and rupture-prone plaques in patients and *ex vivo* studies. The detection of AGE and dicarbonyl compounds might be a novel screening tool for high-risk coronary atherosclerosis in older athletes.

**Methods:**

Concentrations of three different AGE and the dicarbonyl compounds methylglyoxal, glyoxal, and 3-deoxyglucosone were measured in plasma with ultraperformance liquid chromatography tandem mass spectrometry in athletes from the Measuring Athletes’ Risk of Cardiovascular Events 2 study cohort. Coronary plaques, plaque characteristics (calcified, noncalcified or mixed), and coronary artery calcium (CAC) scores were assessed with coronary computed tomography, and potential associations with AGE and dicarbonyl compounds were analyzed using linear and logistic regression.

**Results:**

A total of 289 men were included (60 [quartiles 1–3 = 56–66] yr old, body mass index = 24.5 [22.9–26.6] kg·m^−2^), with a weekly exercise volume of 41 (25–57) MET-hours. Coronary plaques were detected in 241 participants (83%), with a dominant plaque type of calcified plaques in 42%, noncalcified plaques in 12% and mixed plaques in 21%. No AGE or dicarbonyl compounds were associated with total number of plaques or any of the plaque characteristics in adjusted analyses. Similarly, AGE and dicarbonyl compounds were not associated with CAC score.

**Conclusions:**

Concentrations of plasma AGE and dicarbonyl compounds do not predict the presence of coronary plaques, plaque characteristics or CAC scores, in middle-age and older athletes.

Cardiovascular disease (CVD) is the leading cause of mortality worldwide and is dominated by coronary artery disease (CAD) (1). The underlying cause, coronary atherosclerosis, becomes more frequent with advancing age and affects more than 40% of the middle-age general population (2,3). Coronary atherosclerosis has a strong association with lifestyle, and regular physical activity improves cardiovascular health and reduces mortality. However, several studies have demonstrated a U-shaped relationship, with increased coronary atherosclerosis among the most active individuals partaking in high-volume, high-intensity endurance training (4–8). The clinical implications of these findings are still largely unknown (9).

The hallmark of CAD is the development of nonobstructive or obstructive atherosclerotic plaques in coronary arteries. Typical symptoms of chronic plaques include chest pain, shortness of breath, and new onset of heart failure symptoms (10), but a large proportion remains asymptomatic (11). Coronary plaque rupture or erosion can cause plaque destabilization and acute coronary syndromes (10,12), which is the most common cause of sudden cardiac death in middle-age and older athletes (13). To identify high-risk individuals, ESC guidelines recommend the risk prediction models SCORE2 (14) and SCORE2-OP (15), but these algorithms have not been validated in athletes. Many active athletes have more coronary atherosclerosis than the general population, but with a different plaque composition consisting of fewer mixed plaques and more calcified plaques (6). Calcified plaques are considered less rupture prone and are associated with a better prognosis compared with mixed plaques (16), and it could explain why having coronary atherosclerosis is not associated with the same risk in athletes compared with the general population (17). When CAD is suspected or documented in athletes, ESC sports cardiology guidelines recommend the same diagnostic workup as used for the general population (18). However, because of the different plaque composition, in addition to an increased coronary flow reserve (8), these algorithms might not be appropriate and possibly causes unnecessary worry and unwarranted diagnostic tests.

Advanced glycation end products (AGE) are a heterogeneous family of modified proteins and lipoproteins that are generated during normal metabolism and aging. AGE are produced by a nonenzymatic attachment of reducing sugars to proteins, and the production is significantly increased in the presence of hyperglycemia, oxidative stress, and inflammation (19). The accumulation of AGE in blood vessels causes vascular stiffness, generation of foam cells, vascular smooth muscle apoptosis, and vascular inflammation, contributing to atherosclerosis and calcification (19). Several studies have demonstrated that higher concentrations of AGE in plasma predict both the presence and the severity of CAD (20,21) and all-cause and CVD mortality (22). The concentrations of AGE and its dicarbonyl precursors in atherosclerotic plaques have also been demonstrated to predict rupture-prone phenotypes (23); however, it has so far not been examined if this is also the case for plasma concentrations of AGE and dicarbonyl compounds.

The aim of this study, therefore, was to assess the association between plasma AGE and dicarbonyl compounds with the presence of coronary plaques and different plaque characteristics in middle-age and older amateur athletes. We hypothesized that concentrations of plasma AGE and dicarbonyls would be higher among individuals with coronary plaques, and particularly rupture-prone mixed plaques, indicating that these markers could be used in the screening of atherosclerosis and high-risk phenotypes in athletes.

## METHODS

### Study population

We used data from the Measuring Athletes’ Risk of Cardiovascular Events 2 (MARC-2) Study (24) cohort, a 6-yr follow-up study of subclinical coronary atherosclerosis in middle-age and older male athletes. Competitive and recreational amateur athletes 45 yr or older were included in the original MARC-1 study if they were asymptomatic, had a normal sports medical examination, and did not have renal impairment or known CVD (25). Participants who received coronary stents during follow-up of MARC-1 (*n* = 2) or previously had experienced symptoms of contrast reaction (*n* = 5) were excluded from the current substudy, as this would preclude assessment of the coronary computed tomography angiography (CCTA) or make CCTA contraindicated. Follow-up data were collected between May 2019 and February 2020. The Dutch minister of health, welfare, and sport approved the study (1456153-184955-PG), and all participants provided written informed consent before participation. The study was conducted according to the Declaration of Helsinki.

### Exercise characteristics

A detailed description of training volume and training load calculations has previously been published (25). In short, exercise characteristics were collected with a validated questionnaire (26), including information on the duration, intensity, and frequency of sports performed. Different sports were assigned a metabolic equivalent of task (MET) score using the Compendium of Physical Activity (27) and multiplied with volume (reported time spent performing each respective sport) to get the total training load per sport, which was used to calculate the overall total and weekly average training load. In the current substudy, only the average weekly training load during the 6 yr of follow-up is reported.

### AGE and dicarbonyl compounds

Nonfasting venous blood was drawn from all participants, and EDTA plasma was separated and stored at −80°C until analysis. We measured both the protein-bound and the free forms of the AGE Nε-(carboxymethyl)-lysine, Nε-(1-carboxyethyl)-lysine, and Nδ-(5-hydro-5-methyl-4-imidazolon-2-yl)-ornithine, and their dicarbonyl precursors methylglyoxal, glyoxal, and 3-deoxyglucosone, using stable isotope dilution ultraperformance liquid chromatography tandem mass spectrometry (UPLC MS/MS), with a run-to-run time of 8 min. Before measurements, protein-bound AGE were deproteinized with trifluoroacetic acid and free AGE with a mixture of methanol and acetonitrile (1:3, by volume), and then derivatized with 1-butanol:HCl (3:1, v/v). Dicarbonyls were deproteinized using perchloric acid and derivatized with *o*-phenylenediamine. This method of UPLC MS/MS is considered state of the art and has previously been described in detail (28,29). The protein-bound AGE were adjusted for the concentration of lysine in plasma.

### Cardiac computed tomography and assessment of coronary plaques

All participants underwent an electrocardiographic gated cardiac CT scan using a 256-slice CT scanner (Philips Healthcare, Best, The Netherlands) (30). A noncontrast CT was first performed to calculate the coronary artery calcium (CAC) score, followed by a CCTA. CT scans were processed, interpreted, and reported according to guidelines by experienced personnel (30,31). CAC score was calculated with the Agatston method, which aggregates all areas (mm^2^) with density above 130 HU in all CT slices multiplied by a factor reflecting the respective areas’ maximum plaque attenuation: 130–199 HU = 1, 200–299 HU = 2, 300–399 HU = 3, and ≥400 HU = 4. CAC score was stratified into four categories: 0/1–99/100–399/≥400 (32). Coronary plaques were defined as any structure >1 mm^2^ located within the vessel wall and subclassified according to plaque characteristics as calcified, noncalcified, or mixed (partially calcified, both calcified and noncalcified characteristics within the same plaque) (33). In each participant, a coronary plaque characteristic was defined as predominating if it constituted more than 50% of the total number of plaques. Obstructive CAD was defined as >50% stenosis in any coronary artery.

### Statistical analysis

The normality of all variables has been visually assessed for skewness and kurtosis using histograms and QQ-plots, and all biomarkers were transformed by the natural logarithm before analyses because of a right-skewed distribution. Continuous variables are presented as mean ± SD or median (interquartile range) and compared with the Student’s *t*-test or Mann–Whitney *U*-test depending on the normality of distribution. For the comparison of more than two groups, we used ANOVA and the Kruskal–Wallis test. Dichotomous variables are presented as absolute numbers (percentage) and compared using the chi-square test. Associations between the biomarkers and the presence of any plaque or plaque characteristics were assessed with logistic regression. To reduce the number of false positives due to multiple testing, we controlled the false discovery rate using the Benjamini–Hochberg procedure (34), where we accepted a false discovery rate of up to 25%. Correlations between biomarker concentrations and baseline characteristics, CAC score, and training load were assessed with linear regression. We used two-tailed tests and considered *P* values <0.05 significant. All analyses were performed with Stata (version 16; StataCorp LLC, College Station, TX), and figures were made using GraphPad Prism (version 9.3.1; GraphPad Software, San Diego, CA).

## RESULTS

### Patient characteristics

A total of 284 men were included in the current study, and their baseline characteristics are presented in Table 1; 236 participants (83%) had coronary artery plaques with a median CAC score of 55 (7–184) and 14% had obstructive coronary stenoses affecting >50% of the lumen. Stratified by predominating plaque characteristics (see Supplemental Table 1, Supplemental Digital Content, Baseline characteristics grouped by predominating plaque characteristics, http://links.lww.com/MSS/C821), we found that participants with calcified plaques were older, with median age 62.8 (quartiles 1–3 = 57.8–68.3) yr compared with 59.2 (55.9–61.1) yr for noncalcified plaques and 61.5 (58.0–65.9) yr for mixed plaques (*P* for trend 0.011), and that participants with predominantly calcified plaques had a higher training load at 44.3 (29.2–58.3) MET-h·wk^−1^ compared with 35.9 (18.5–50.8) MET-h·wk^−1^ for noncalcified plaques and 41.8 (26.4–56.6) MET-h·wk^−1^ for mixed plaques (*P* for trend 0.042).

**TABLE 1:** Baseline characteristics grouped by the presence of unstratified plaques.

	Total	No Plaques	Plaques	*P*
	*Ν* = 284	*n* = 48	*n* = 236	
Age, yr	60.0 (56.3–66.0)	57.7 (55.1–61.3)	60.6 (56.7–66.1)	0.008
BMI, kg·m^−2^	24.5 (22.9–26.7)	24.0 (22.4–25.2)	24.7 (23.1–26.8)	0.024
Systolic BP, mm Hg	140 ± 18	135 ± 15	140 ± 18	0.057
Current smoker, *n* (%)	9 (3%)	1 (2%)	8 (3%)	0.64
Total cholesterol, mmol·L^−1^	5.2 ± 0.9	5.2 ± 0.8	5.2 ± 0.9	0.86
Hypertension, *n* (%)	36 (13%)	5 (10%)	31 (13%)	0.61
Diabetes mellitus, *n* (%)	9 (3%)	2 (4%)	7 (3%)	0.67
CVD, *n* (%)	6 (2%)	1 (2%)	5 (2%)	0.99
Family history of CVD, *n* (%)	100 (35%)	9 (19%)	91 (39%)	0.009
CAC score, Agatston units	31 (0–132)	0 (0–0)	55 (7–184)	<0.001
Obstructive CAD, *n* (%)	32 (11%)	0 (0%)	32 (14%)	0.007
MET·h·wk^−1^	40.5 (25.9–56.6)	38.5 (21.3–61.3)	40.7 (26.8–56.4)	0.69
AGE				
Free CML, nmol·L^−1^	112 (90–135)	117 (92–141)	110 (89–134)	0.56
Free CEL, nmol·L^−1^	58 (48–72)	57 (47–76)	58 (48–72)	0.75
Free MG-H1, nmol·L^−1^	329 (220–465)	278 (218–478)	332 (220–449)	0.50
CML, nmol·L^−1^	3378 (3023–3720)	3387 (3140–3644)	3373 (3012–3726)	0.88
CEL, nmol·L^−1^	1599 (1337–1910)	1595 (1356–1818)	1599 (1326–1918)	0.77
MG-H1, nmol·L^−1^	1237 (1106–1383)	1215 (1142–1371)	1242 (1100–1383)	0.71
Dicarbonyl compounds				
MGO, nmol·L^−1^	439 (387–491)	433 (392–471)	440 (385–497)	0.62
GO, nmol·L^−1^	516 (456–592)	492 (425–547)	520 (464–600)	0.046
3-DG, nmol·L^−1^	1201 (1123–1268)	1222 (1163–1280)	1197 (1120–1264)	0.23

Data are presented as mean ± SD, median (interquartile range), or *n* (%).

BMI, body mass index; BP, blood pressure; MET, metabolic equivalent; CML, Nε-(carboxymethyl)-lysine; CEL, Nε-(1-carboxyethyl)-lysine; MG-H1, Nδ-(5-hydro-5-methyl-4-imidazolon-2-yl)-ornithine; MGO, methylglyoxal; GO, glyoxal; 3-DG, 3-deoxyglucosone (3-DG).

### AGE and dicarbonyl compounds and association with coronary plaques

**FIGURE 1:**
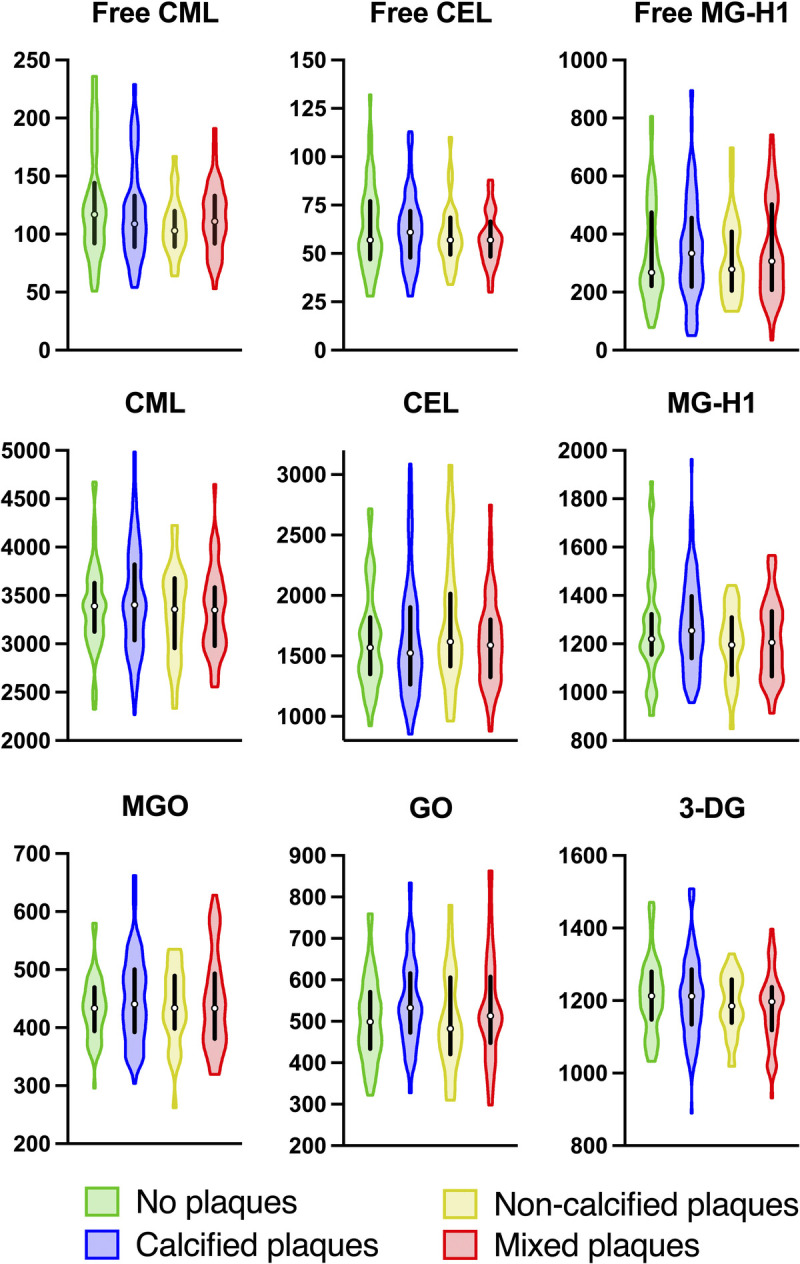
Concentrations of AGE and dicarbonyl compounds stratified by predominating plaque characteristic. Distribution of concentrations demonstrated by violin plots with median concentrations (quartiles 1–3) marked with *black lines*. We found no differences in biomarker concentrations after stratifying participants by predominating plaque characteristics. CML, Nε-(carboxymethyl)-lysine; CEL, Nε-(1-carboxyethyl)-lysine; MG-H1, Nδ-(5-hydro-5-methyl-4-imidazolon-2-yl)-ornithine; MGO, methylglyoxal; GO, glyoxal; 3-DG, 3-deoxyglucosone (3-DG).

Median concentrations of AGE and dicarbonyl compounds are presented Table 1, and their associations with baseline characteristics can be found in Supplemental Table 2 (see Supplemental Digital Content, Variables associated with increasing concentrations of free AGEs, protein-bound AGEs and dicarbonyl compounds, http://links.lww.com/MSS/C821). Calcified plaques were the predominating plaque characteristic in 100 participants (42% of participants with plaques), followed by mixed plaques in 50 participants (21%) and noncalcified plaques in 30 participants (13%). Concentrations of plasma AGE and dicarbonyl compounds were not significantly different between participants with or without coronary plaques (Table 1) or between the different predominating plaque characteristic groups (Fig. 1). Correspondingly, concentrations of plasma AGE and dicarbonyl compounds did not predict the presence of any of the different plaque characteristics in logistic regression (Table 2). Glyoxal concentrations correlated with calcified plaques in univariate regression (*P* = 0.047), but after controlling the false discovery rate, this correlation was attenuated and no longer significant.

**TABLE 2:** Associations between biomarkers and presence of different plaque characteristics.

	Calcified Plaques (*n* = 184)		Noncalcified Plaques (*n* = 124)		Mixed Plaques (*n* = 175)	
	OR (95% CI)	*P*	OR (95% CI)	*P*	OR (95% CI)	*P*
AGE						
_ln_Free CML, nmol·L^−1^	1.06 (0.52–2.18)	0.87	0.86 (0.43–1.73)	0.68	1.23 (0.60–2.50)	0.57
_ln_Free CEL, nmol·L^−1^	1.34 (0.62–2.87)	0.46	1.02 (0.49–2.11)	0.96	1.38 (0.65–2.93)	0.40
_ln_Free MG-H1, nmol·L^−1^	0.99 (0.66–1.49)	0.97	0.79 (0.53–1.17)	0.24	1.16 (0.78–1.73)	0.46
_ln_CML, nmol·L^−1^	1.64 (0.34–7.93)	0.54	0.37 (0.08–1.70)	0.20	0.72 (0.15–3.35)	0.67
_ln_CEL, nmol·L^−1^	0.98 (0.40–2.42)	0.96	1.30 (0.55–3.12)	0.55	1.01 (0.41–2.45)	0.99
_ln_MG-H1, nmol·L^−1^	2.18 (0.64–7.43)	0.21	0.76 (0.25–2.30)	0.62	0.65 (0.22–1.97)	0.45
Dicarbonyl compounds						
_ln_MGO, nmol·L^−1^	2.07 (0.58–7.36)	0.26	0.45 (0.14–1.52)	0.20	1.06 (0.37–3.06)	0.91
_ln_GO, nmol·L^−1^	3.50 (1.04–11.81)	0.043	0.83 (0.27–2.60)	0.76	2.13 (0.66–6.89)	0.21
_ln_3-DG, nmol·L^−1^	0.29 (0.02–3.35)	0.32	0.36 (0.03–3.96)	0.41	0.49 (0.04–5.52)	0.57

After controlling the false discovery rate using the Benjamini–Hochberg procedure, none of the associations were significant.

Abbreviations as described in Table 1.

### AGE and dicarbonyl compounds and association with CAC score

**FIGURE 2:**
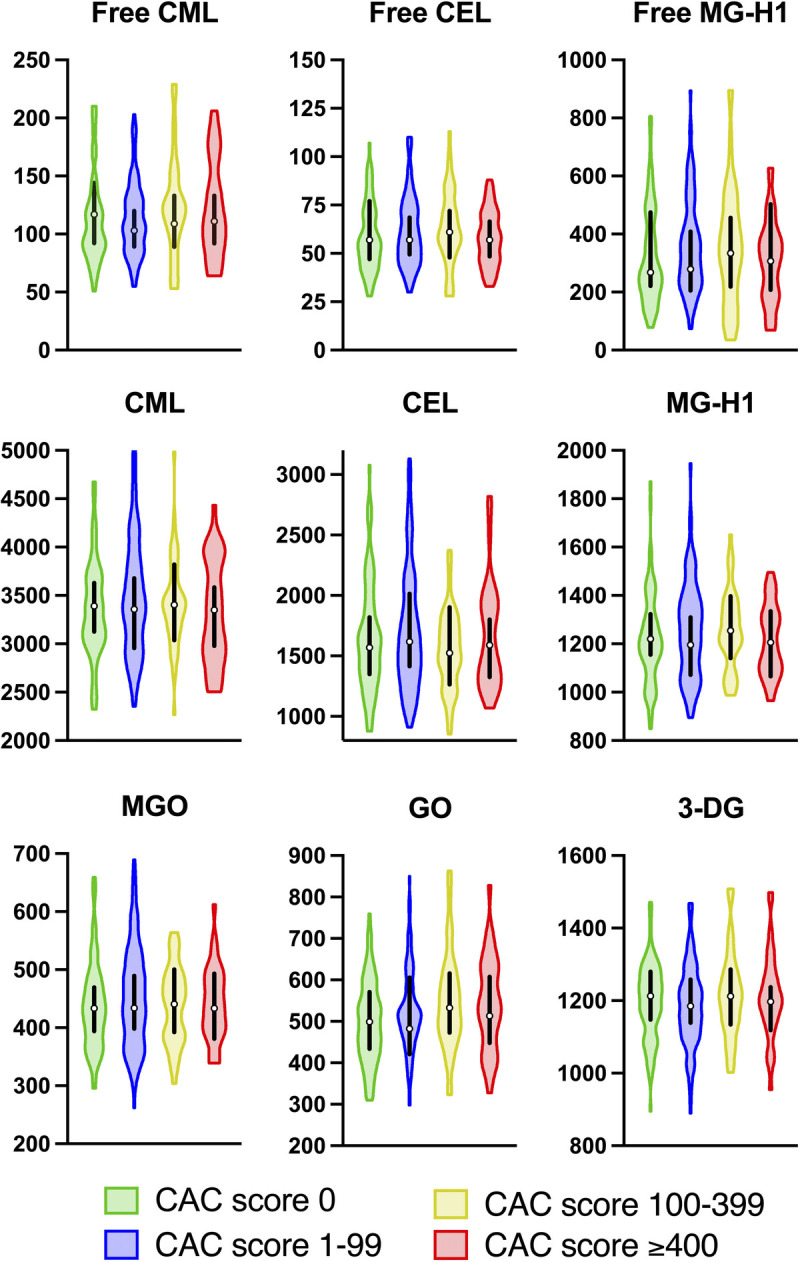
Concentrations of AGE and dicarbonyl compounds stratified by CAC score groups. Distribution of concentrations demonstrated by violin plots with median concentrations (quartiles 1–3) marked with *black lines*. We found no differences in biomarker concentrations after stratifying participants by CAC score groups. CML, Nε-(carboxymethyl)-lysine; CEL, Nε-(1-carboxyethyl)-lysine; MG-H1, Nδ-(5-hydro-5-methyl-4-imidazolon-2-yl)-ornithine; MGO, methylglyoxal; GO, glyoxal; 3-DG, 3-deoxyglucosone (3-DG).

In the total study cohort, the median CAC score was 31 (0–132), with 82 (29%) participants scoring 0, 115 (40%) scoring between 1 and 99, 50 (18%) scoring between 100 and 399, and 37 (13%) scoring ≥400. CAC score as a continuous variable was not correlated with any of the free AGE, protein-bound AGE, or dicarbonyl compounds in unadjusted regression analysis (see Supplemental Table 2, Supplemental Digital Content, Variables associated with increasing concentrations of free AGEs, protein-bound AGEs and dicarbonyl compounds, http://links.lww.com/MSS/C821). Similarly, there were no differences in concentrations of AGE or dicarbonyl compounds when stratified according to CAC score groups (Fig. 2 and Supplemental Table 3, Supplemental Digital Content, Median concentrations of free AGEs, protein-bound AGEs and dicarbonyl compounds stratified by CAC score groups, http://links.lww.com/MSS/C821).

## DISCUSSION

We assessed the association of AGE and dicarbonyl compounds with coronary atherosclerosis and plaque characteristics to evaluate whether these biomarkers can be used to screen for atherosclerosis or rupture-prone plaques in middle-age and older athletes. We found that plasma concentrations of free AGE, protein-bound AGE, and dicarbonyl compounds did not correlate with the total number of coronary artery plaques or with any of the groups of predominating plaque characteristics. In line with this, there were no associations between AGE and dicarbonyl compounds with CAC score or CAC score categories. These findings indicate that plasma AGE and dicarbonyl compounds cannot be used to identify coronary atherosclerosis in athletes.

The number of middle-age and older endurance athletes is increasing (35,36), including disciplines such as marathon, triathlon, and long-distance cycling. As high-volume high-intensity training is associated with increasing coronary atherosclerosis, but with a different plaque composition suggesting a better prognosis (37), novel ways to screen for CAD in athletes are warranted (17). Using CVD risk prediction algorithms (14,15) and diagnostic workup algorithms (10) intended for the general population could lead to a diagnostic cascade with associated risks in athletes with low risk of developing serious disease. As demonstrated by the ISCHEMIA trial (38), current practice of revascularization of stable coronary disease is at best questionable and should be reserved for certain subpopulations. For middle-age and older athletes, where coronary atherosclerosis is not associated with the same risks as in the general population (4), a more conservative approach seems appropriate and alternative risk assessment tools are needed.

Excessive formation of AGE and dicarbonyl compounds is linked to aging, the development of a range of different diseases, and mortality (19). Recently, an increasing number of studies have emphasized their role in the development of CAD by contributing to arterial stiffness, vasodilation, and atherogenesis (39). Angiographic studies have demonstrated correlations between plasma AGE and number of significantly stenosed vessels (20), implying that AGE could provide a new target for screening and detecting patients with high-risk CVD. However, most previous studies have consisted of patients with high or very high probability of CAD.

In our cohort of middle-age and older athletes, concentrations of AGE or dicarbonyl compounds measured in plasma did not predict the presence of coronary plaques or different plaque characteristics. This contradicts previous findings which showed that associations between AGE and CAD (20,21) and concentrations of AGE measured in tissue are associated with rupture-prone atherosclerotic plaques (23). The cause of this discrepancy is likely linked to the difference in burden and phenotype of CAD among participants. In contrast to previous studies (20–22), our cohort was predominantly healthy, with only 2% having established CVD, a low burden of comorbidities and known risk factors of CVD, and only 14% of participants with coronary plaques detectable on CCTA having obstructive disease. It is possible that one or more of CVD risk factors contribute as mediators of AGE production. The composition of plaque characteristics in our cohort was similar to other cohorts of athletes, with a high degree of calcified plaques (6). The fact that calcified plaques have less active inflammation than other plaque phenotypes (40) could explain the lack of associations found in our study, as inflammation is a known promoter of the formation of AGE (19). In addition to this, regular physical exercise training in general has an anti-inflammatory effect, which further could reduce inflammation in coronary plaques in athletes and consequently diminish AGE formation. Despite our findings, it could still be that concentrations of AGE within plaques depend on plaque phenotypes in athletes, as has been demonstrated in patients with more CVD risk factors and comorbidities (23), but that this association is not reflected by plasma measurements. It is also possible that a study of AGE in athletes with higher plaque burden and more rupture-prone plaques would have yielded different results.

### Strengths and limitations

Strengths of our study include the assessment of coronary plaques and CAC scores using CCTA in combination with measurements of AGE and dicarbonyl compounds using UPLC MS/MS in a large cohort of athletes. Measurements of AGE and dicarbonyl compounds were done as a batch to avoid variation in laboratory calibration over time. A limitation of our study is the low degree of significant CAD. Although 83% of participants had coronary plaques detectable on CCTA, only 14% had obstructive disease, and as plaques were defined as any structure >1 mm^2^ located within the vessel wall, many of these patients could have had minimal and not clinically relevant plaques. Plaque burden instead of the number of plaques might have yielded different results and should be tested in future studies. Lastly, our study cohort only consisted of men, of which 99% identified as white, which limits the generalizability of our findings.

## CONCLUSIONS

In our cohort of middle-age and older amateur athletes, plasma concentrations of AGE and dicarbonyl compounds were not associated with the presence of coronary plaques, plaque characteristics, or CAC scores and cannot be used to screen for CAD in older athletes. It will have to be tested in future studies if AGE and dicarbonyl compounds can predict CAD in other low-risk cohorts.
